# Hyperphosphatemia in an 11-year-old girl with acute myeloid leukemia: Answers

**DOI:** 10.1007/s00467-018-4101-5

**Published:** 2018-10-05

**Authors:** Monique Albersen, Arend Bökenkamp, Hans Schotman, Stephanie Smetsers

**Affiliations:** 1 Amsterdam UMC, Department of Clinical Chemistry, Vrije Universiteit Amsterdam, De Boelelaan 1117, 1081 HV Amsterdam, The Netherlands; 2 Amsterdam UMC, Department of Pediatric Nephrology, Vrije Universiteit Amsterdam, De Boelelaan 1117, 1081 HV Amsterdam, The Netherlands; 3 Amsterdam UMC, Department of Pediatric Oncology, Vrije Universiteit Amsterdam, De Boelelaan 1117, 1081 HV Amsterdam, The Netherlands; 4 Prinses Máxima Centrum voor Kinderoncologie, Heidelberglaan 25, 3584 CS Utrecht, The Netherlands

## Commentary

Serum phosphate concentration is mainly affected by dietary intake and renal excretion of phosphate. Phosphate homeostasis is regulated by the phosphaturic hormones FGF23 and parathyroid hormone, as well as by growth hormone and vitamin D. Other sources of phosphate include leakage of intracellular phosphate during tumor lysis, rhabdomyolysis, or hemolysis or a transcellular shift of phosphate during diabetic ketoacidosis or lactate acidosis.

The differential diagnosis of hyperphosphatemia can be divided into four major groups: (i) increased phosphate intake, (ii) transcellular phosphate shift, (iii) diminished phosphate excretion, and (iv) pseudohyperphosphatemia.

The patient in this case received no dietary phosphate supplements or phosphate-containing laxatives. The underlying malignancy was in remission and there were no signs of rhabdomyolysis or hemolysis (Table 1). There was also no transcellular phosphate shift by diabetic ketoacidosis or lactic acidosis.

**Table 1:** Additional laboratory investigations in this patient

Laboratory parameter	Concentration	Reference	Day of admittance
Potassium	*3.3*	3.6–4.8 mmol/L	7
Calcium	2.30	2.25–2.75 mmol/L	12
Urea	3.7	3.0–7.5 mmol/L	8
Creatinine	*35*	39–60 μmol/L	8
Hemoglobin	*5.2*	6.5–10.0 mmol/L	7
Bilirubin	20	< 20 μmol/L	8
PTH	1.6	< 10 pmol/L	21
Triglycerides	1.5	< 2.0 mmol/L	15
ASAT	8	< 51 U/L	8
Lactate	1.0	< 2.2 mmol/L	8
Glucose	6.9	< 7.8 mmol/L*	8

Concentrations below the reference are shown in italic. *Random glucose measurement

Laboratory investigations excluded renal insufficiency, and tubular reabsorption of phosphate was normal (87%; reference 85–95%) [1]. Hypoparathyroidism leading to increased tubular reabsorption of phosphate was ruled out. Growth analysis showed no signs of growth hormone excess, although growth hormone concentration was not measured.

Having excluded all in vivo causes, pseudohyperphosphatemia, i.e., an artifact during the measurement of phosphate, was considered. This in vitro phenomenon has been reported for immunoglobulins, hyperlipidemia, and bilirubin (reviewed by Liamis et al. [2]). Although there is an ongoing debate on the clinical relevance of some of these interferences [3], the influence of elevated levels of paraproteins by Waldenstrom’s macroglobulinemia and multiple myeloma is well established [2]. Also, sample hemolysis is known to interfere with the laboratory phosphate assay [2].

In our case, triglyceride and bilirubin concentrations were normal, as were immunoglobulins. Samples from this patient were non-hemolytic. Analysis was therefore extended to medications known to cause pseudohyperphosphatemia, such as heparin and tissue plasminogen activator [2]. Another drug which has been linked to interference of the laboratory phosphate assay is liposomal amphotericin B (AmBisome®), an antimycotic antibiotic [2, 4, 5]. In our patient, hyperphosphatemia was first noted two days after liposomal amphotericin B had been prescribed for treatment of pulmonary aspergillosis. Therefore, this drug was considered the most probable culprit of the hyperphosphatemia.

In the liposomal preparation of amphotericin B, the drug is packed in liposomes. This has greatly reduced its nephrotoxicity (reviewed by Stone et al. [6] and Adler-Moore et al. [7]). Besides several molecules of amphotericin B, the spherical liposomes contain phosphatidylcholine and distearoyl phosphatidylglycerol [8]. AmBisome® is thought to exert its effect by binding to the fungal cell wall, after which amphotericin B particles are released, causing pore formation in the fungal cell membrane, generation of reactive oxygen species, and ion leakage, ultimately resulting in fungal cell death [6, 7].

In order to support our hypothesis, two plasma samples of the patient and remnant plasma samples of four controls were analyzed before and after ultra-filtration using Amicon® Ultra 10k membrane filters. These filters have a molecular weight cutoff of 10 kDa, meaning that molecules with a weight > 10 kDa (corresponding to a diameter > 2.84 nm for spherical protein molecules [9]) are retained by the filter membrane. Although they are not protein molecules, we hypothesize that liposomes in the AmBisome® formulation (with a diameter of 45–80 nm [8]) will be retained by the filter membrane, prohibiting their subsequent interference with the laboratory phosphate assay.

Ultra-filtration was performed using a Hettich mikro 22r centrifuge (30 min at a speed of 20,160*g*) and phosphate concentrations were determined before and after ultra-filtration by colorimetric analysis using ammonium phosphomolybdate in the presence of sulfuric acid [10]. In this assay, plasma phosphate forms a complex with ammonium molybdate at low pH, which is achieved by the addition of sulfuric acid. Absorption of the phosphomolybdate complex is determined spectrophotometrically at 340 nm and is directly related to the phosphate concentration present in the sample (mmol/L).

It is hypothesized that either acid-induced hydrolysis of phosphate present in the lipid bilayer of the liposomes [4] or an amphotericin B-mediated increase in formation of the phosphomolybdate complex [5] is responsible for the pseudohyperphosphatemia observed in patients receiving AmBisome® treatment, provided that, like in our laboratory, phosphate is determined using assays based on the principle described above.

The results of the ultrafiltration experiments are shown in Table 2. In line with the reports of Lane and Jensen [4, 5], phosphate concentrations were unaffected by ultra-filtration in control samples (difference ranging from − 3.1 to + 2.8%), but there was a clear reduction of plasma phosphate in the patient samples (− 14.9 and − 13.6%, respectively).

**Table 2:** Effect of filtration on plasma phosphate in the patient and four controls

Samples		Plasma phosphate (mmol/L)		
		Before filtration	After filtration	Difference (%)
Patient samples	1	2.28	1.94	− 0.34 (− 14.9%)
	2	1.91	1.65	− 0.26 (− 13.6%)
Control samples	1	1.27	1.23	− 0.04 (− 3.1%)
	2	1.56	1.52	− 0.04 (− 2.6%)
	3	1.08	1.11	+ 0.03 (+ 2.8%)
	4	1.40	1.40	± 0.00 (± 0.0%)

Patient samples 1 and 2 were withdrawn on days 15 and 19 of admittance, respectively

The phosphate concentration in the first patient sample, however, did not fall below the upper age-related reference limit of 1.80 mmol/L [11]. Here, diurnal variation and the influence of dietary intake could have played a role [12]. After discontinuation of AmBisome® several months later, plasma phosphate in this patient returned to normal (1.29 mmol/L).

In conclusion, liposomal amphotericin B may lead to falsely elevated serum phosphate concentrations when phosphate is determined using the phosphomolybdate method. In this way, AmBisome® can mask true hypophosphatemia or falsely suggest hyperphosphatemia. Although true hyperphosphatemia is unlikely when renal function and calcium concentration are normal, it is important to recognize the possibility of pseudohyperphosphatemia to avoid unnecessary interventions aiming at treating this in vitro phenomenon. The presence of pseudohyperphosphatemia can be determined by ultra-filtration, which will result in reduction of plasma phosphate.

### Answer

Pseudohyperphosphatemia
